# Correlation between elastic modulus and clinical severity of pathological scars: a cross-sectional study

**DOI:** 10.1038/s41598-021-02730-0

**Published:** 2021-12-02

**Authors:** Jing Hang, Jie Chen, Weixin Zhang, Tao Yuan, Yang Xu, Bingrong Zhou

**Affiliations:** 1 Department of Ultrasound, The First Affiliated Hospital of Nanjing Medical University, Nanjing, 210029 China; 2 Department of Ultrasound, Nanjing Hospital of Chinese Medicine Affiliated to Nanjing University of Chinese Medicine, Nanjing, 210029 China; 3 Department of Dermatology, The First Affiliated Hospital of Nanjing Medical University, Nanjing, 210029 China

**Keywords:** Skin diseases, Skin manifestations

## Abstract

Though widely used to assess pathological scars, the modified Vancouver Scar Scale (mVSS) is neither convenient nor objective. Shear wave elastography (SWE) is used to evaluate the stiffness of pathological scars. We aimed to determine the correlation between mVSS score and elastic modulus (EM) measured by SWE for pathological scars. Clinical information including ultrasound (US) results of the enrolled patients with pathological scars was analyzed. The clinical severity of the pathological scars was evaluated by mVSS. Skin stiffness, as represented by EM, was calculated using SWE. The average EM of the whole scar (EM_WHOLE_), hardest part of the scar (EM_HARDEST_), and normal appearance of the skin around the scar (EM_NORMAL_) were also recorded. Enrolled in this study were 69 pathological scars, including 28 hypertrophic scars and 41 keloids. The univariable regression analyses showed that the EM of pathological scars was closely related to mVSS score, while the linear multivariable regression analyses showed no significantly correlation. Curve fitting and threshold effect analysis revealed that when EM_WHOLE_ was less than 166.6 kPa or EM_HARDEST_ was less than 133.07 kPa, EM was positively correlated with mVSS score. In stratified analysis, there was no significant linear correlation and threshold effect between EM_WHOLE_ and mVSS score in hypertrophic scars or keloids. However, the fully adjusted smooth curves presented a linear association between mVSS score and EM_HARDEST_ in keloids (the adjusted β [95% CI] was 0.010 [0.001, 0.018]), but a threshold and nonlinear association were found in hypertrophic scars. When EM_HARDEST_ was less than 156.13 kPa, the mVSS score increased along with the hardest scar part stiffness; the adjusted β (95% CI) was 0.024 (0.009, 0.038). In conclusion, EM of pathological scars measured by SWE were correlated with mVSS within a threshold range, and showed different association patterns in hypertrophic scars and keloids.

Each year, approximately 1 billion people worldwide develop pathological scars, including keloids and hypertrophic scars^1^. The scar tissue of the keloid usually overgrows the original boundary of the wound. Most keloid patients have a genetic predisposition and dark skinned people are more likely to develop keloids^2^. Unlike keloids, hypertrophic scars do not exceed the original wound boundary and may subside after a few months^3^. Pathological scars not only cause itching, pain, and other subjective discomfort, but also raise the patient’s mental and psychological concerns over aesthetics. In severe cases, scars can even result in deformities and body dysfunction. Many studies have shown that skin tension, abnormal biological function of scar fibroblasts (an imbalance between extracellular matrix synthesis and degradation), vascular dysplasia, disordered cytokine regulation, and immunological factors are closely related to the occurrence of scars^4–6^.

The current treatment for pathological scars mainly includes surgical resection, radiotherapy, intralesional injection, and laser ablation. Accurate evaluation is important to the diagnosis of pathological scars and assessment of therapeutic effects. Biopsy, the most accurate method, cannot be widely applied due to its invasive nature. Other comprehensive evaluation methods, such as the modified Vancouver scar scale (mVSS)^7^, have many disadvantages. mVSS evaluates scar severity in terms of thickness, vascularity, pliability, pigmentation, pain and pruritus^8^. However, using mVSS to evaluate pathological scars mainly depends on the subjective experience of clinicians, and requires more than two doctors, making it quite inconvenient^9^.

Several studies have reported that high-frequency ultrasound (US) can be used to observe the clinical activity of pathological scars^10–12^. Ultrasonic elastography, a new method to detect tissue stiffness, falls into two types: strain elastography (SE) and shear wave elastography (SWE)^13^. SWE is a non-invasive and quantitative imaging technique^14^ using the acoustic radiation force pulse to produce shear waves on the body. Tissue stiffness is calculated by measuring the propagation velocity of shear wave in the tissue, which quantitatively reflects tissue elasticity^15^. Aya R reported using SE and SWE techniques to evaluate the therapeutic effect in three keloid cases^16,17^. They believed that ultrasonic elastography could be a potential quantitative tool to assess pathological scar activity. Guo R^12^ recently reported for the first time that the average shear wave velocity of a keloid (C_mean_) was positively correlated with VSS, keloid thickness, and blood flow. Using the formula E = 3ρC^2^, the elastic modulus (EM) value (E) was obtained, where C was the shear wave velocity and ρ was the density. Therefore, both C_mean_ and EM can evaluate tissue stiffness. However, in that study, only the hardest lesion in the thickest part of the keloid was measured. Furthermore, cases of hypertrophic scars were not included, and most of the cases enrolled were from Western China. DeJong H et al. used SWE to measure velocity of hypertrophic burn scars and found high correlations between the measured velocity and VSS pliability sub-scores^18^. Gender, injury time, body location, and Fitzpatrick skin type all demonstrated significant associations with velocity^18^. Zuccaro J et al. used acoustic radiation force impulse ultrasound elastography to quantify the stiffness of hypertrophic burn scars, but no correlations were noted between EM and VSS score^19^. Up to now, only a few studies have investigated whether EM can be used to evaluate the clinical activity of pathological scars, and their conclusions are inconsistent. In the present study, we analyzed the clinical information and SWE ultrasonic images of 69 pathological scars and found a threshold and nonlinear association between skin stiffness and mVSS score of pathological scars. Besides, the correlation between SWE stiffness and mVSS showed different patterns for hypertrophic scars and keloids.

## Results

**Figure 1:**
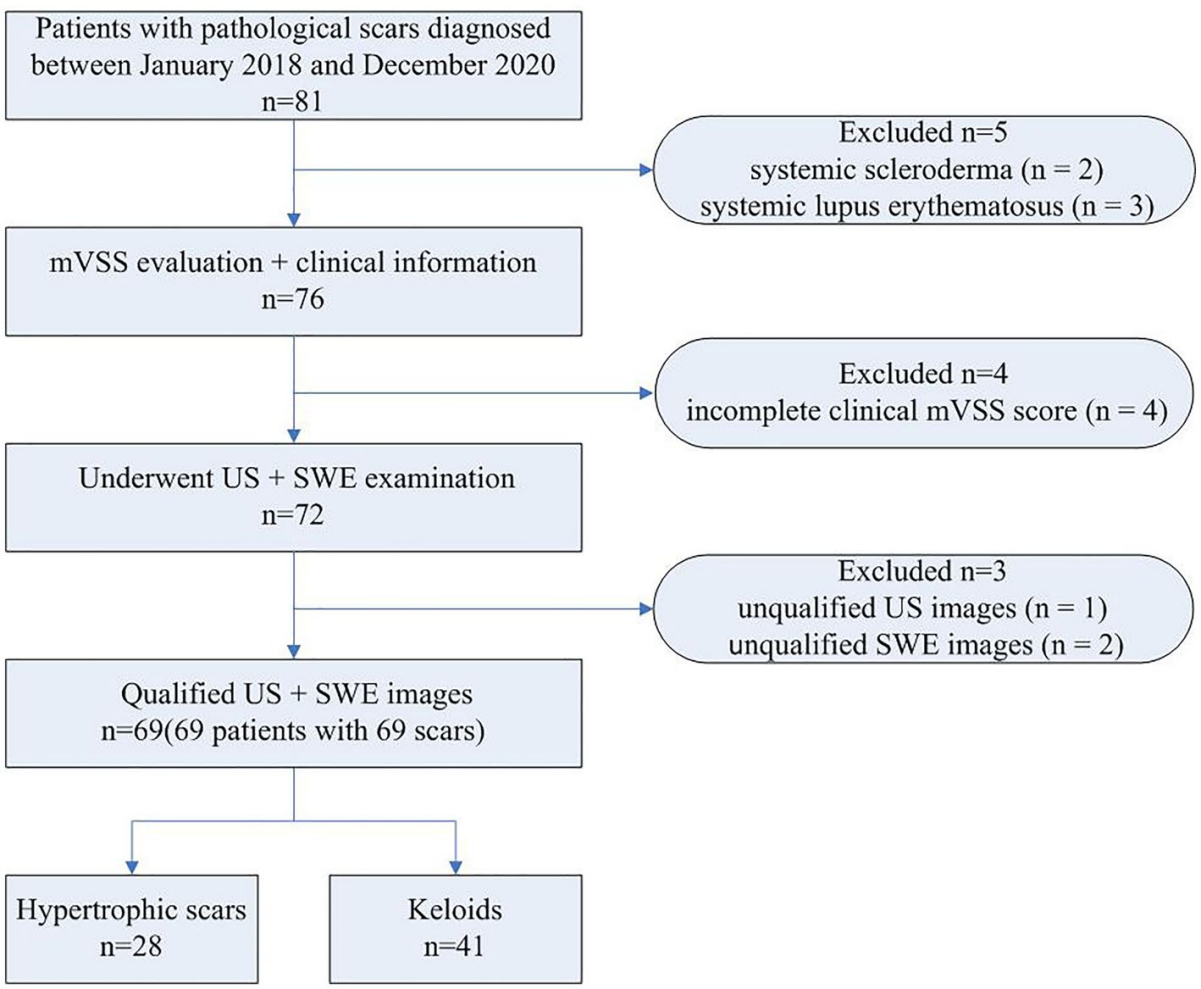
Inclusion criteria and experiment procedure of the study.

**Figure 2:**
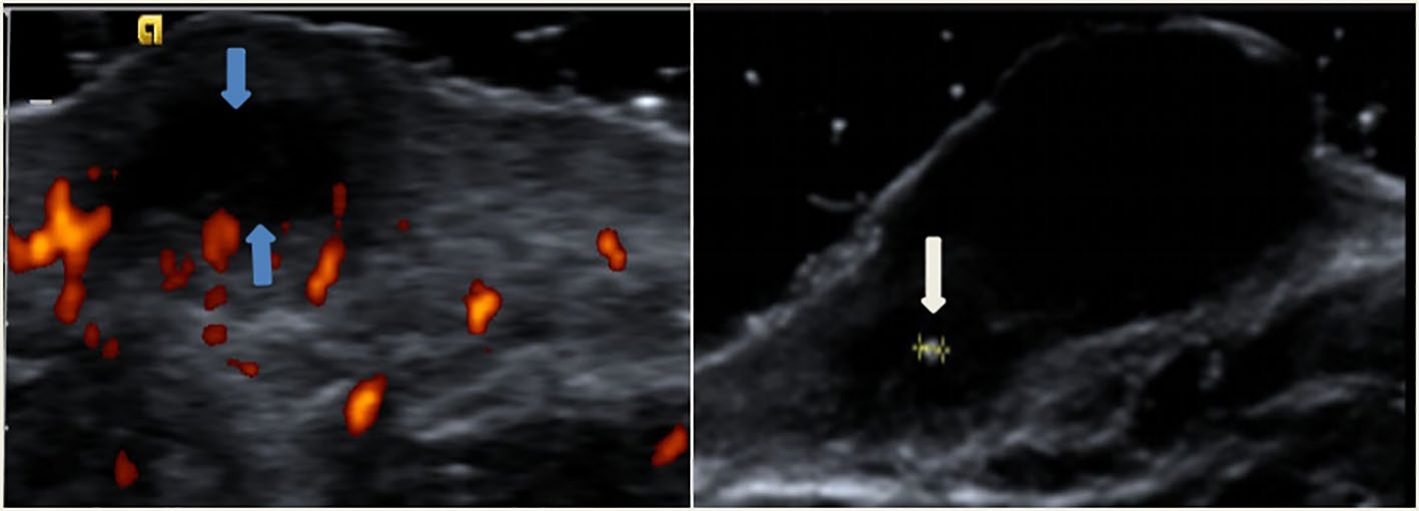
A keloid at the lip in a 37-year-old female patient. Blue arrow indicates the fistula of pathological scar; white arrow indicates the calcification (1 mm) of the pathological scar.

The study flowchart is illustrated in Fig. 1. A total of 69 patients with 69 scars (28 hypertrophic scars and 41 keloids) were recruited. The baseline clinical and demographic characteristics of the included patients are shown in Table 1. The patients’ average age was 33.30 ± 14.88 years; 32 were male (46.38%) and 37 were female (53.62%). The median scar thickness, supracutaneous height, and intradermal height were 4.60 mm, 1.70 mm, and 2.70 mm, respectively. Thirty-nine scars were in the chest and abdomen (56.52%). Arterial blood flow was detected in 27 scars (39.13%), and 27 scars had peripheral blood flow patterns (39.13%). Three scars had calcification (4.35%) (one of which is shown in Fig. 2), while 3 scars had fistula (4.35%).

**Table 1:** Demographic and clinical characteristics of 69 pathological scars.

Characteristics	Study cohort
Age, years, mean ± SD	33.30 ± 14.88
Duration, years, median (Q1–Q3)	4.00 (2.00–8.00)
mVSS, mean ± SD	9.24 ± 3.02
Scar length, mm, median (Q1–Q3)	26.00 (17.10–35.80)
Scar width, mm, median (Q1–Q3)	12.80 (9.80–16.40)
Scar thickness, mm, median (Q1–Q3)	4.60 (3.50–6.10)
Supracutaneous height, mm, median (Q1–Q3)	1.70 (1.10–2.50)
Intradermal height, mm, median (Q1–Q3)	2.70 (1.90–3.80)
EM_WHOLE_, kPa, median (Q1–Q3)	129.60 (75.70–198.47)
EM_HARDEST_, kPa, median (Q1–Q3)	183.10 (111.47–286.53)
EM_NORMAL_, kPa, median (Q1–Q3)	29.60 (19.70–48.40)
**Gender**	
Male, no. (%)	32 (46.38%)
Female, no. (%)	37 (53.62%)
**Etiology**	
Spontaneity, no. (%)	26 (37.68%)
Inflammation, no. (%)	18 (26.09%)
Operation, no. (%)	16 (23.19%)
Chemical, no. (%)	9 (13.04%)
**Treatment history**	
Untreated, no. (%)	36 (52.17%)
Hormone, no. (%)	16 (23.19%)
Laser, no. (%)	3 (4.35%)
Operation, no. (%)	7 (10.14%)
Others, no. (%)	2 (2.90%)
Mixed, no. (%)	5 (7.25%)
**Scar location**	
Head and neck, no. (%)	10 (14.49%)
Limbs, no. (%)	8 (11.59%)
Chest and abdomen, no. (%)	39 (56.52%)
Back, no. (%)	12 (17.39%)
**Echogenicity**	
Uniform echo, no. (%)	13 (18.84%)
Mixed echo, no. (%)	56 (81.16%)
**Boundary**	
Clear, no. (%)	56 (81.16%)
Unclear, no. (%)	13 (18.84%)
**Infiltration level**	
Dermis, no. (%)	65 (94.20%)
Subcutaneous fat layer, no. (%)	4 (5.80%)
**Subclinical fistulous tract**	
Absent, no. (%)	66 (95.65%)
Present, no. (%)	3 (4.35%)
**Calcification**	
Absent, no. (%)	66 (95.65%)
Present, no. (%)	3 (4.35%)
**Blood flow type**	
Absent, no. (%)	10 (14.49%)
Vein, no. (%)	8 (11.59%)
Artery and vein, no. (%)	24 (34.78%)
Artery, no.(%)	27 (39.13%)
**Blood flow distribution pattern**	
Absent, no. (%)	11 (15.94%)
Central type, no. (%)	14 (20.29%)
Mixed type, no. (%)	17 (24.64%)
Peripheral type, no. (%)	27 (39.13%)
**Alder**	
No blood flow, no. (%)	27 (39.13%)
I, no. (%)	15 (21.74%)
II, no. (%)	16 (23.19%)
III, no. (%)	11 (15.94%)
**Scar type**	
Hypertrophic scar, no. (%)	28 (40.58%)
Keloid, no. (%)	41 (59.42%)

The univariable analysis showed that EM_WHOLE_ and EM_HARDEST_ were correlated with mVSS, and the β (95% CI) were 0.020 (0.012–0.029) and 0.016 (0.011–0.022), respectively (Table 2). Besides age, length, width, thickness, supracutaneous and intradermal heights, mixed echo, absent of blood flow, absent of blood flow patterns, Alder grade, and the type of the pathological scar were also associated with mVSS. Compared with scar with peripheral blood flow, the scars absent of blood flow showed a relative lower mVSS score (β = -3.167, 95% CI [-5.139, -1.195], *P* = 0.002).

**Table 2:** Univariable analysis of the clinical factors associated with mVSS in 69 pathological scars.

Characteristics	mVSS	*P* value
Age, years	0.059 (0.013, 0.106)	0.014
Duration, years	0.045 (−0.073, 0.163)	0.459
Scar length, mm	0.091 (0.055, 0.126)	< 0.00001
Scar width, mm	0.189 (0.094, 0.283)	0.00021
Supracutaneous height, mm	1.262 (0.821, 1.702)	< 0.00001
Intradermal height, mm	0.569 (0.156, 0.982)	0.009
Scar thickness, mm	0.594 (0.345, 0.843)	0.00001
EM_WHOLE_	0.020 (0.012, 0.029)	0.00003
EM_HARDEST_	0.016 (0.011, 0.022)	< 0.00001
EM_NORMAL_	0.012 (−0.007, 0.031)	0.204
**Gender**		
Male	Reference	
Female	−0.545 (−1.977, 0.887) 0.45851	0.459
**Treatment**		
Untreated	Reference	
Glucocorticoid	1.132 (−0.632, 2.896)	0.213
Laser	−2.931 (−6.459, 0.597)	0.108
Operation	1.355 (−1.070, 3.780)	0.278
Others	0.569 (−3.696, 4.835)	0.794
Mixed	0.269 (−2.532, 3.071)	0.851
**Scar location**		
Head and neck	Reference	
Limbs	−0.563 (−3.324, 2.199)	0.691
Chest and abdomen	1.436 (−0.628, 3.500)	0.177
Back	−0.042 (−2.535, 2.451)	0.974
**Echogenicity**		
Uniform echo	Reference	
Mixed echo	2.759 (1.048, 4.470)	0.002
**Boundary**		
Clear	Reference	
Unclear	0.748 (−1.077, 2.573)	0.425
**Infiltration depth**		
Dermis	Reference	
Subcutaneous fat layer	2.798 (−0.196, 5.793)	0.071
**Subclinical fistulous tracts**		
Absent	Reference	
Present	0.970 (−2.539, 4.478)	0.590
**Calcifications**		
Absent	Reference	
Present	1.144 (−2.362, 4.650)	0.525
**Blood flow type**		
Artery	Reference	
Vein	−0.562 (−3.204, 2.079)	0.678
Artery and vein	0.438 (−1.659, 2.534)	0.684
Absent	−2.167 (−4.228, −0.105)	0.043
**Blood flow distribution pattern**		
Peripheral type	Reference	
Central type	−1.643 (−3.864, 0.578)	0.152
Mixed type	−0.765 (−2.898, 1.369)	0.485
Absent	−3.167 (−5.139, −1.195)	0.002
**Alder**		
No blood flow	Reference	
I	1.667 (−0.106, 3.440)	0.070
II	2.198 (0.461, 3.935)	0.016
III	3.348 (1.379, 5.318)	0.001
**Scar type**		
Hypertrophic scar	Reference	
Keloid	2.386 (1.042, 3.730)	0.001

**Figure 3:**
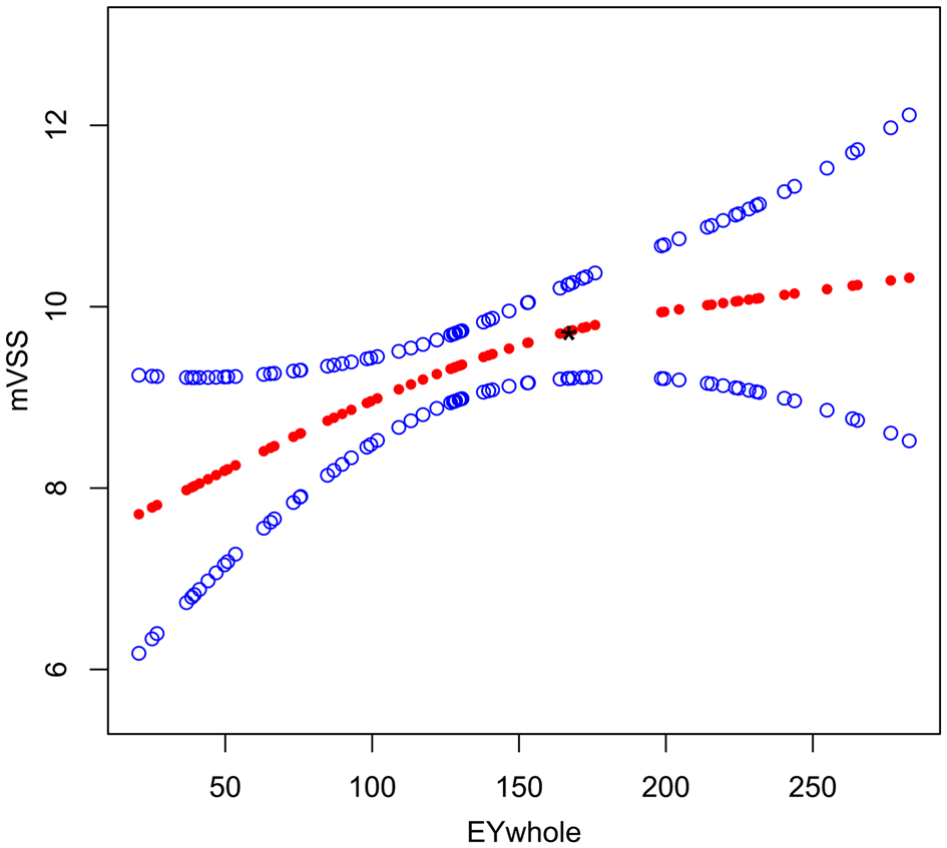
Adjusted smoothing function of the threshold effect analysis of the relationship between EM_WHOLE_ and mVSS. *represents the threshold point (166.6 kPa). Adjusted for: age; duration; etiology; treatment; scar location; scar length; scar width; echogenicity; subclinical fistulous tracts; supracutaneous height; intradermal height; blood flow type; blood flow distribution pattern; scar type.

**Figure 4:**
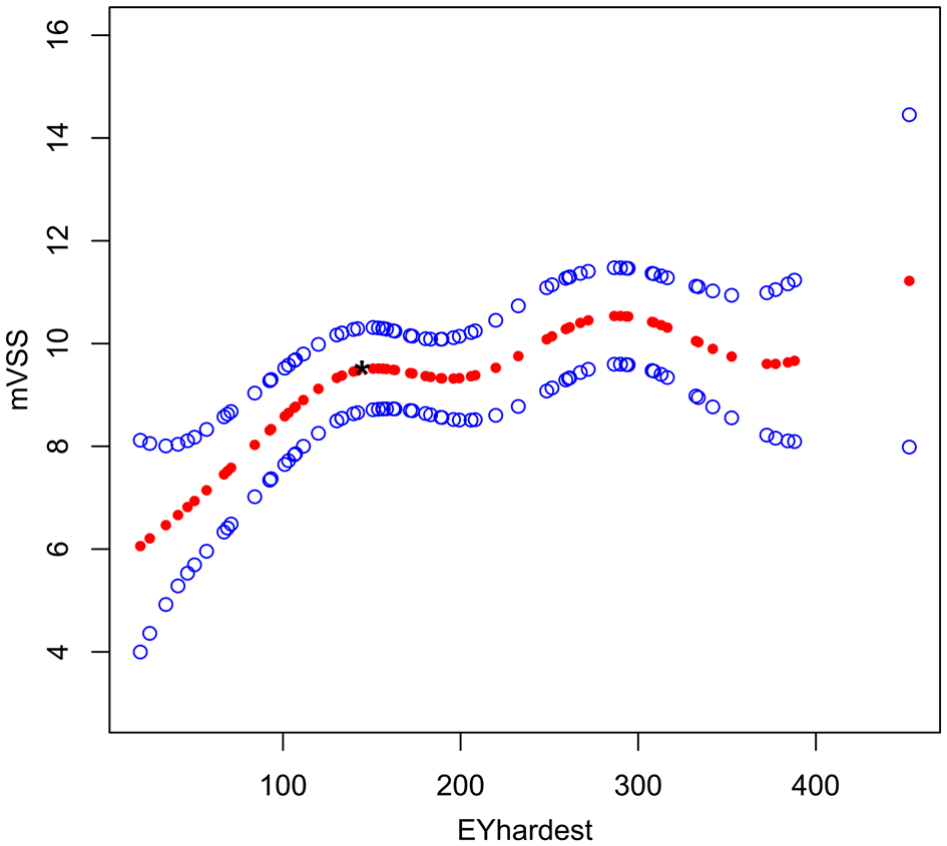
Adjusted smoothing function of the threshold effect analysis of the relationship between EM_HARDEST_ and mVSS. * represents the threshold point (133.07 kPa). Adjusted for: age; duration; etiology; scar location; scar length; scar width; echogenicity; subclinical fistulous tracts; supracutaneous height; blood flow type; scar type.

As shown in Table 3, in the linear multivariable regression analysis, if cofounders were adjusted, the EM (EM_WHOLE_ and EM_HARDEST_) of the scar was not related to mVSS, i.e., the adjusted β (95% CI) was 0.010 (-0.001, 0.021) and 0.008 (0.002, 0.014), respectively. The results of the univariable and multivariable regression analyses were not consistent. In the smooth curve fitting model, we found that there was a threshold effect between EM of pathological scar and mVSS. When EM_WHOLE_ was less than 166.6 kPa, the mVSS increased by 0.020 for each additional 1 kPa increase in EM_WHOLE_; the adjusted β (95% CI) was 0.020 (0.004, 0.036) (Table 4, Fig. 3). When EM_WHOLE_ was higher than 166.6 kPa, the mVSS value decreased as EM_WHOLE_ increased, and the adjusted β (95% CI) was -0.004 (-0.023, 0.015). However, the change was not statistically significant (*P* = 0.677). When EM_HARDEST_ was less than 133.07 kPa, the mVSS increased by 0.031 for each 1 kPa increase in the hardest scar part stiffness; the adjusted β (95% CI) was 0.031 (0.012, 0.050) (Table 4, Fig. 4). When EM_HARDEST_ was greater than 133.07 kPa, the mVSS did not change significantly as EM_HARDEST_ increased; the adjusted β (95% CI) was 0.003 (-0.004, 0.010) (*P* = 0.382).

**Table 3:** Multivariable regression analysis of the EY_WHOLE_ and EY_HARDEST_ associated with mVSS in 69 pathological scars.

Exposure	Non−adjusted	Adjust I	Adjust II
**EM**_**WHOLE**_** group**			
EM_WHOLE_ (20.6–130.2)	Reference	Reference	Reference
EM_WHOLE_ (130.6–408.0)	2.501 (1.198, 3.804), *P* = 0.0004	0.997 (−0.525, 2.520), *P* = 0.206	0.740 (−0.945, 2.426), *P* = 0.395
EM_WHOLE_ group trend	0.020 (0.012, 0.029), *P* < 0.00001	0.010 (−0.001, 0.021), *P* = 0.095	0.009 (−0.002, 0.020), *P* = 0.126
Exposure	Non-adjusted	Adjust I*	Adjust II*
**EM**_**HARDEST**_** group**			
EM_HARDEST_ (19.7–188.9)	Reference	Reference	Reference
EM_HARDEST_ (188.9–554.5)	2.704 (1.424, 3.984), *P* = 0.00010	0.924 (−0.403, 2.252), *P* = 0.178	1.304 (−0.299, 2.907), *P* = 0.118
EY_HARDEST_ group trend	0.016 (0.011, 0.022), *P* < 0.00001	0.008 (0.002, 0.014), *P* = 0.015	0.009 (0.002, 0.016), *P* = 0.017

Adjust I model is adjusted for: age; duration; etiology; treatment; scar location; scar length; scar width; echogenicity; subclinical fistulous tracts; supracutaneous height; intradermal height; blood flow type; blood flow distribution pattern; scar type.

Adjust II model is adjusted for: age; duration; etiology; treatment; scar location; scar length; scar width; echogenicity; infiltration level; subclinical fistulous tracts; supracutaneous height; intradermal height; blood flow type; blood flow distribution pattern; Alder; scar type.

Adjust I* model is adjusted for: age; duration; etiology; scar location; scar length; scar width; echogenicity; subclinical fistulous tracts; supracutaneous height; blood flow type; scar type.

Adjust II* model is adjusted for: age; duration; etiology; scar location; scar length; scar width; echogenicity; infiltration level; subclinical fistulous tracts; supracutaneous height; blood flow type; blood flow distribution pattern; Alder; scar type.

**Table 4:** Threshold effect analysis of the correlation between EM_WHOLE_, EM_HARDEST_ and mVSS in 69 pathological scars.

	mVSS
**EM**_WHOLE_*	
EM_WHOLE_ ≤ 166.6	0.020 (0.004, 0.036), *P* = 0.019
EM_WHOLE_ > 166.6	−0.004 (−0.023, 0.015), *P* = 0.677
**EM**_**HARDEST**_**#**	
EM_HARDEST_ ≤ 133.07	0.031 (0.012, 0.050), *P* = 0.002
EM_HARDEST_ > 133.07	0.003 (−0.004, 0.010), *P* = 0.382

*Adjusted variables: age; duration; etiology; treatment; scar location; scar length; scar width; echogenicity; subclinical fistulous tracts; supracutaneous height; intradermal height; blood flow type; blood flow distribution pattern; scar type.

^#^Adjusted variables: age; duration; etiology; scar location; scar length; scar width; echogenicity; subclinical fistulous tracts; supracutaneous height; blood flow type; scar type.

**Figure 5:**
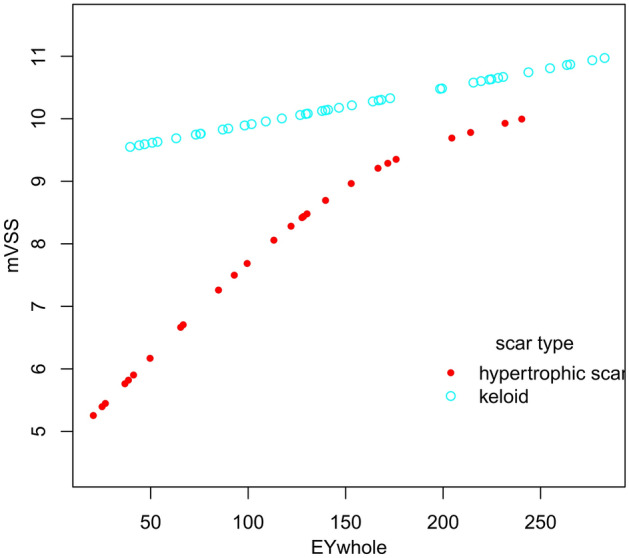
The stratified analysis of adjusted smoothing function of the threshold effect analysis of the relationship between EM_WHOLE_ and mVSS. Adjusted for: age; duration; etiology; treatment; scar location; scar length; scar width; echogenicity; subclinical fistulous tracts; supracutaneous height; intradermal height; blood flow type; blood flow distribution pattern.

Tables 5 and Fig. 5 present stratified association between EM_WHOLE_ and mVSS in keloids or hypertrophic scars. A non-linear relationship between EM_WHOLE_ and mVSS in keloids or hypertrophic scars was demonstrated as logarithmic likelihood ratio test (LRT) results showed that *P* < 0.05 for both conditions. The turning point in a two-piecewise regression model between EM_WHOLE_ and mVSS in the hypertrophic scar were 84.8 kPa. Despite whether EM_WHOLE_ was greater or less than the turning point, the mVSS score increased with the increase of EM_WHOLE_. However, after adjusting for confounding factors, the statistical significance was not found. The similar results were also obtained in keloids.

**Table 5:** Subgroup analysis of threshold effect analysis between EM_WHOLE_ and mVSS in 69 pathological scars.

Scar type	Hypertrophic scar	Keloid
Model I (linear analysis)	0.024 (0.004, 0.044), *P* = 0.097	0.021 (0.004, 0.038), *P* = 0.031
**Model II (two-piecewise regression models)**		
Turning point	84.8	89.8
< Turning point	0.062 (0.026, 0.097), *P* = 0.076	0.082 (0.004, 0.159), *P* = 0.059
> Turning point	0.016 (0.001, 0.031), *P* = 0.166	0.017 (−0.000, 0.034), *P* = 0.071
LRT test	< 0.001	0.01

Data are presented as β (95%CI) P-value. Model I, linear analysis; Model II, non-linear analysis; LRT test, logarithmic likelihood ratio test. (p value < 0.05 means Model II is significantly different from Model I, which indicates a non-linear relationship). Adjusted for: age; duration; etiology; treatment; scar location; scar length; scar width; echogenicity; subclinical fistulous tracts; supracutaneous height; intradermal height; blood flow type; blood flow distribution pattern.

**Figure 6:**
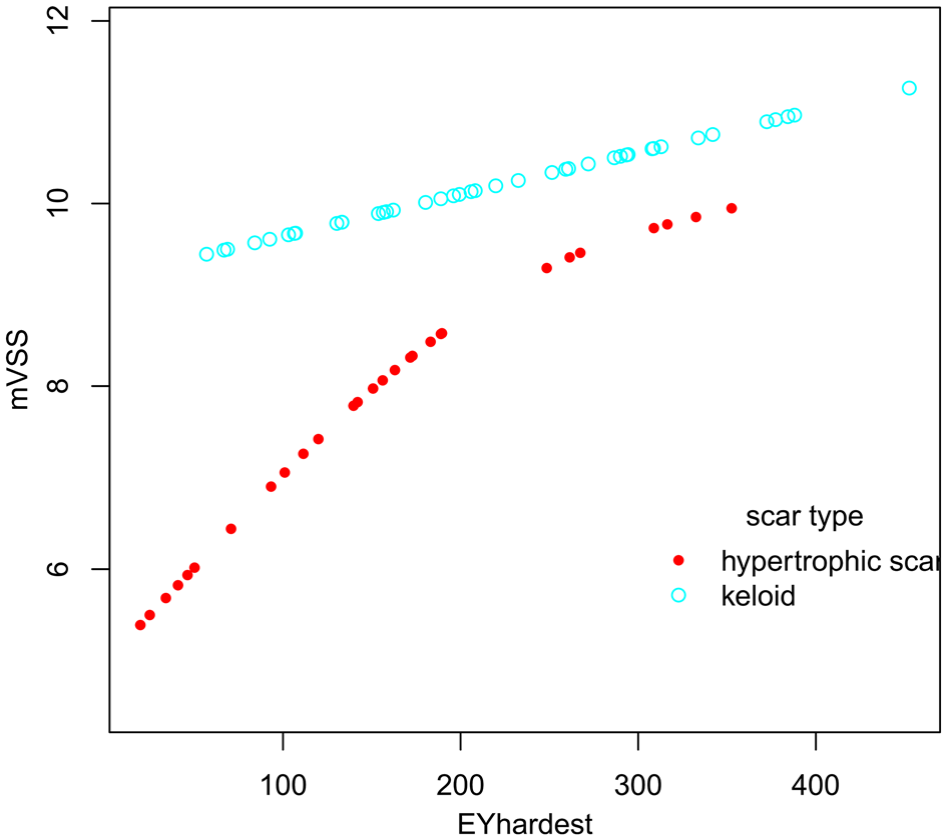
The stratified analysis of adjusted smoothing function of the threshold effect analysis of the relationship between EM_HARDEST_ and mVSS. Adjusted for: age; duration; etiology; scar location; scar length; scar width; Echogenicity; subclinical fistulous tracts; supracutaneous height; blood flow type.

Tables 6 and Fig. 6 present stratified association between EM_HARDEST_ and mVSS in keloids or hypertrophic scars. A non-linear relationship between EM_HARDEST_ and mVSS was demonstrated in hypertrophic scars. The turning point in a two-piecewise regression model between EM_HARDEST_ and mVSS in hypertrophic scars was 156.13 kPa. When EM_HARDEST_ was less than 156.13 kPa, the mVSS increased by 0.024 for each 1 kPa increase in the hardest scar stiffness; the adjusted β (95% CI) was 0.024 (0.009, 0.038). When EM_HARDEST_ was greater than 156.13 kPa, the mVSS did not change significantly as EM_HARDEST_ increased; the adjusted β (95% CI) was -0.003 (-0.017, 0.011) (*P* = 0.731). In keloids, LRT results showed p = 0.07, which demonstrated a linear relationship between EM_HARDEST_ and mVSS. The mVSS value increased as EM_HARDEST_ increased and the adjusted β (95% CI) was 0.010 (0.001, 0.018) (*P* = 0.042).

**Table 6:** Subgroup analysis of threshold effect analysis between EM_HARDEST_ and mVSS in 69 pathological scars.

Scar type	Hypertrophic scar	Keloid
Model I (linear analysis)	0.010 (−0.001, 0.021), *P* = 0.108	0.010 (0.001, 0.018), *P* = 0.042
**Model II (two-piecewise regression models)**		
turning point	156.13	133.07
< turning point	0.024 (0.009, 0.038), *P* = 0.013	0.038 (−0.004, 0.080), *P* = 0.090
> turning point	−0.003 (−0.017, 0.011), *P* = 0.731	0.007 (−0.003, 0.016), *P* = 0.185
LRT test	< 0.001	0.07

Data are presented as β (95%CI) P-value. Model I, linear analysis; Model II, non-linear analysis. LRT test, logarithmic likelihood ratio test. (p value < 0.05 means Model II is significantly different from Model I, which indicates a non-linear relationship). Adjusted for: age; duration; etiology; scar location; scar length; scar width; echogenicity; subclinical fistulous tracts; supracutaneous height; blood flow type.

In addition, the correlation between EM values (EM_WHOLE,_ EM_HARDEST_) and mVSS score components (pigmentation, vascularity, pliability, thickness, pain, pruritus) was analyzed (Supplementary table 1). The correlation coefficient between EM_WHOLE_ and all mVSS score components showed statistical significance (*P* < 0.05), except for vascularity and pain. The correlation coefficient between EM_HARDEST_ and mVSS score components (vascularity, pliability, thickness, pruritus) showed statistical significance (*P* < 0.05).

## Discussion

The clinical activity evaluation of pathological scars is important for choosing treatment modalities and monitoring therapeutic effects. However, mVSS, the most commonly used evaluation method in clinical practice, is based on the subjective assessment of the physicians^20^. In the present study, we found a threshold effect between scar EM values (EM_WHOLE_ and EM_HARDEST_) and mVSS. Besides, there are different correlation patterns of scar stiffness and mVSS in hypertrophic scars and keloids.

This study found that high-frequency US could accurately measure the supracutaneous scar height and there was a correlation between supracutaneous height and mVSS as reported by previous studies^20–22^. Due to severe inflammation or even suppuration in pathological scars, fistula and calcification could form^23^. Lobos N et al. reported that US could be used to observe subclinical features of keloids such as internal fistula, calcification, and peripheral nerve swelling^10^. In this study, we also observed sinuses in some pathological scars. In addition, we found that US can detect the blood flow inside the scar, including arteries and veins. The existence of blood flow in the scar was related to the scar activity, and the scar with blood flow showed a higher level of clinical activity, which is consistent with the results of previous studies^10^. These results demonstrated that US had high repeatability and reliability in the evaluation of pathological scars.

SWE elastography is a new noninvasive method for stiffness evaluation. Skin stiffness measured by SWE can be used to evaluate the severity of scleroderma, portwine stains, and other skin conditions^24,25^. Using SWE technology to assess keloids, some studies found EM could objectively measure the pliability of scars^16,17^. In burn scar patients, the shear wave velocity measured by SWE can be used to discriminate between scars and normal skin and assess scar severity^18^. In this study, our data showed that the EM was correlated with several sub-indexes in mVSS, such as pliability, thickness, pigmentation and pruritus, and the correlation coefficient between stiffness and pliability was the highest. Therefore, there exists a direct correlation between EM values and mVSS. However, after adjusting some clinical variables, we found that there was no “inherent” linear correlation between EM and mVSS. These results indicate the complexity of scar activity evaluation. Similarly, Zuccaro J et al. performed acoustic radiation force impulse ultrasound elastography in 16 pediatric patients with hypertrophic scars, and found that the correlation with VSS could not be observed by using the scar stiffness value alone^19^.

Through curve fitting and threshold effect analysis, we found that the correlation between EM_WHOLE_, EM_HARDEST_ and mVSS did not show a linear correlation, but there was a threshold effect, that is, EM_WHOLE_ and EM_HARDEST_ were only correlated with mVSS score within a certain threshold range. The pathological scar formation after wound healing can generally be divided into the “inflammatory, proliferative and regression” stages. In the inflammatory and proliferative stages, microvessels and fibroblasts accumulate in wounded skin, and collagen gradually deposits around the microvessels^26^. At this time, the thickness and stiffness of the skin obviously increase along with the appearance of subjective symptoms such as pain and itching^27^. Therefore, the mVSS score increases, and the EM stiffness measured by SWE elevates accordingly. In the regression stage, squeezed by the surrounding accumulated collagen and fibroblasts, the microvessels in the scar are partially or completely occluded^28^, meanwhile pruritus, pain, and other discomfort are also reduced, leading to the decrease of mVSS score. However, at the regression stage, the proportion of collagen in pathological scar changes. In the inflammation and proliferation stages, the proportion of collagen III increases, changing from 20 to 50%^29^. With the gradual maturity of the pathological scar, the type I collagen proportion and the collagen fibrosis increase. As a result, the stiffness of pathological scar in regression stage usually does not decrease significantly^30^. We believe that this mechanism may explain the threshold effect of the correlation between pathological scar stiffness and mVSS score. However, whether this threshold indicates the progression of pathological scar to late proliferation or early regression still needs further investigation.

Interestingly, in the stratified analysis, we found that EM_HARDEST_ could better reflect the activity of pathological scars than EM_WHOLE_. Hypertrophic scars mostly follow the evolution process of “inflammation-proliferation-regression”, so the correlation between EM and scar activity showed the above mentioned threshold effects. But the mechanisms underlying the evolution of keloids are complex. The latest guideline divides keloids into “tumor type” and “inflammatory type” according to their origin^31–34^. “Inflammatory type” keloids usually have predominant inflammatory response inside the scar tissues, while for “tumor type” keloids, the inflammatory reaction is manifested by mild-to-moderate collagen deposition^31–34^. Due to the long-term repeated alternating “inflammation and repair” cycle, most keloids show neoplastic growth behavior^5^. The continuous collagen deposition in keloids makes the collagen bundles denser and forms isolated non-vascular crude collagen accumulation^28^. “Inflammatory type” keloids usually show higher mVSS scores because of vasodilation, pigmentation, and other obvious symptoms. Meanwhile, in the part of the active keloid with a high inflammatory reaction, obvious cellular edema and stiffness are found^31–34^. In some keloid-related studies, collagen in the mature part is more likely to accumulate and form collagen nodules, while proliferative scar tissues are often found in the immature area lacking collagen^23^. Huang C et al. believed that collagen nodules and vitreous degeneration co-existed in keloids, which led to heterogeneity of keloids^33^. This also explains the inconsistent maturity within keloids. Hence, EM_WHOLE_ cannot accurately indicate the overall stiffness of the scar, yet the EM_HARDEST_ value at the hardest part can better reflect the activity of keloids.

This study had several limitations. First, since skin biopsy is invasive and can easily lead to inflammatory reaction and aggravate the activity of pathological scars, the scars included in this study were not examined histologically or pathologically. Therefore, the US imaging and histopathological results of these pathological scars were not compared and their correlation was not explored. Second, the study’s retrospective design may induce unavoidable bias in clinical information. Third, the sample size of this observational study was small and the data were derived from a single center.

In conclusion, our study confirmed for the first time that there is a threshold effect between EM and scar activity in pathological scars, and showed different association patterns for hypertrophic scars and keloids. These results provide useful clinical data of SWE elastic ultrasound in evaluating the activity of pathological scars. The conclusion of this study, if further confirmed in clinical studies with larger sample sizes, will help clinicians to evaluate the severity of pathological scars more objectively.

## Materials and methods

### Patients

We analyzed conventional US and SWE images of patients with pathological scars diagnosed in the Departments of Dermatology and Ultrasound Imaging of the First Affiliated Hospital of Nanjing Medical University between January 2018 and December 2020. The study was approved by the Ethics Committee of the First Affiliated Hospital of Nanjing Medical University (approval number: 2020-SR-271) and abided by the Helsinki Declaration. All patients provided written informed consent prior to the US and SWE examinations. Clinical information such as scar duration, injury cause, previous treatment, and scar location were recorded before US examination. Clinical scar photos were collected and mVSS scores were calculated by two dermatologists. The exclusion criteria were as follows: (1) other skin diseases, such as scleroderma or systemic lupus erythematosus; and (2) incomplete clinical data or US and SWE examination images.

### Clinical evaluation of pathological scars

In this study, two dermatologists with more than 5 years’ experience in scar evaluation applied the mVSS, including pigmentation (0–3), vascularity (0–3), thickness (0–3), pain (0–2) and itching (0–2) and pliability (0–5)^20^. The highest total score was 18 with 0 indicating normal skin, and 18 indicating the most serious skin injury and represented the highest activity of scar. The definitions are listed in Supplementary Table 2. Prior to the mVSS evaluation, the patient’s scar location, scar age, previous treatment history, and etiology were also collected. After the evaluation, 2 dermatologists determined whether it was a hypertrophic scar (the lesion within the original injury boundary) or a keloid (the lesion exceeding the original injury boundary)^31^.

### US examination of pathological scars

Two doctors with more than 5 years’ skin US experience conducted an US examination on all pathological scars enrolled in this study. Information from gray-scale US, color Doppler US and SWE imaging was collected using a real-time US device (Aixplorer, SuperSonic Imagine, Aix-en-Provence, France) equipped with a 4–15 MHz linear array transducer. During the examination, all patients were asked to hold their breath and refrain from other movements for at least 3 s to ensure image stability. Tissue stiffness was quantitatively evaluated and presented as E. In case of tissue elasticity, a larger E indicated the increased stiffness of the tissue.

**Figure 7:**
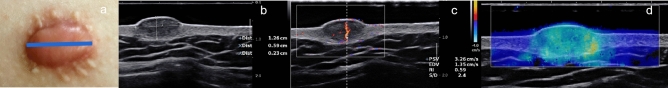
Schematic diagram of the measurement of supracutaneous height. A hypertrophic scar in the right chest from a 23-year-old male patient. (**a**) The gross view and position of probe. (**b**) “ + ” represents the connection of the epidermal layer on both side; “ × ” represents the thickness of the scar; “Υ” represents the supracutaneous height of the scar. The intradermal height of the scar is calculated by subtracting the supracutaneous height from the thickness. (**c**) Artery found in the central part of the scar. (**d**) The SWE image of the scar.

**Figure 8:**
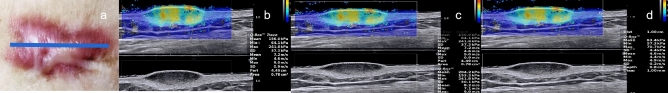
The clinical and SWE images of a keloid in the left chest from a 64-year-old female patient. (**a**) The gross view and position of probe. (**b**) SWE image shows the average EM of the whole scar (EM_WHOLE_) is 156.0 kPa. (**c**) The average EM of the hardest part in the scar (EM_HARDEST_) is 204.2 kPa. (**d**) SWE image shows that the surrounding normal skin in the area one centimeter away from the edge of the scar(EM_NORMAL_) is 63.4 kPa.

The doctors examined the grey-scale US images first and observed scar echogenicity, size, thickness, supracutaneous (Fig. 7) and intradermal height, margins, infiltration level, and presence of calcification or a fistula. Scar blood flow type, distribution, and grade^35^ were also recorded during the color Doppler evaluation. The supracutaneous height was obtained by calculating the distance between the highest point of the scar and the line connecting the epidermal layers on both sides of the scar. Figure 7 is a schematic diagram of the measurement method. On gray-scale US images, a uniform echo was defined as a uniform echo distribution in the scar, while a mixed echo contained different echo distributions. The border between scar and the surrounding normal dermis was defined as the boundary of them. Infiltration level was defined as the involved skin layer of the scar on the US images. In color Doppler US images, according to the blood flow of the arteries or veins inside the scar, we divided the blood flow into the following types: absent, vein, artery and vein, or vein. Based on the region of blood flow distribution in the scar, the blood flow distribution was divided into the following patterns: absent, central, mixed, or peripheral. Due to the anisotropy effect of the scar on elastography^11^, we performed the SWE examination on the longest diameter of pathological scar. An ultrasonic pad (round, 3-mm thickness; Beijing Deji Luzhong Medical Equipment Co., Ltd., Beijing, China) was used between the probe and the skin. When SWE was activated, the transducer was placed perpendicularly to the skin on the ultrasonic pad without compression or movement. A previous study^36^ showed that the sampling area of the region of interest (ROI) affected EM. Our study required a circular ROI to cover more than 50% of the whole scar area. The US doctor select the ROI covering the hardest part of the scar, where the highest EM was measured by the machine. A site about 1 cm away from the edge of the scar was selected to measure the normal skin stiffness. Skin stiffness (EM, kPa) was calculated automatically by the machine. Finally, the average EM value of the whole scar (EM_WHOLE_), hardest part of the scar (EM_HARDEST_), and normal skin (EM_NORMAL_) were calculated and recorded. The inner diameter of the ROI of the hardest part of scar and the surrounding normal skin was 1 cm (Fig. 8). Each group of parameters was measured three times, and the average value was used for the statistical analysis. On SWE mapping, a default chromatic scale with hues from blue to red was representative of soft-to-hard tissue stiffness. The tissue EM was expressed in kPa, and the upper limit of the display was set to 600 kPa. Three or more SWE cine loops that lasted for > 10 s from each lesion were acquired for analysis. The specific definitions are listed in Supplementary Table 3.

### Statistical analyses

The statistical analyses were performed using EmpowerStats (X&Y Solutions, Boston, MA, USA) and R Software (version 3.5.2; R Foundation for Statistical Computing, Vienna, Austria). Normality was evaluated by Kolmogorov–Smirnov test. Continuous normally distributed data were expressed as mean ± standard deviation, continuous skewed data were expressed as median (Q1–Q3), and classified data were shown as number of patients (%). A univariable analysis was performed to detect variables related to the mVSS values, while a multivariable analysis was used to analyze the possible variables that might be related to the mVSS value to determine the relationship between mVSS value and EM of the whole scar and the hardest scar part, and confirm the above relationship by sensitivity analysis. A two-piecewise linear regression model was used to test the threshold effect of mVSS value and EM values of the whole scar and the hardest scar part. Finally, the threshold effect was analyzed to obtain the critical value. The threshold level (or turning point) was determined by repeated experiments, including selecting the turning point along a predetermined interval and then selecting the turning point with the maximum model likelihood. The logarithmic likelihood ratio test was used to compare the linear regression model with a two-part linear model. Finally, spearman correlations were used to calculate the degree of association between mVSS value and EM of the whole scar and the hardest scar part. Correlation ranging between 0.1 to 0.39 was considered weak, 0.4–0.69 moderate, 0.7 to 0.89 strong, and 0.9–1.0 very strong^37^. We also calculated the statistical power to confirm the robustness of the associations. Values of *P* < 0.05 were considered statistically significant.

## Supplementary Information

Supplementary Information 1.

Supplementary Information 2.

Supplementary Information 3.

## Data Availability

All datasets generated for this study can be available from corresponding author at reasonable request.
