# Supplementary material for: Correlation between elastic modulus and clinical severity of pathological scars: a cross-sectional study
Source: Sci Rep. 2021 Dec 2;11:23324. doi: 10.1038/s41598-021-02730-0 (PMC8639709; doi:10.1038/s41598-021-02730-0)
Supplement: Supplementary file 1 — Supplementary Information 1. [file 41598_2021_2730_MOESM1_ESM.docx]

| Supplementary Table 1. Correlation analysis of EM_WHOLE_, EM_HARDEST_ and mVSS score components in 69 pathological scar | | | |
| --- | --- | --- | --- |
| Var 1 | Var 2 | Correlation | P.value |
| Pigmentation | EM_WHOLE_ | 0.298 | 0.013 |
| Pigmentation | EM_HARDEST_ | 0.304 | 0.011 |
| Vascularity | EM_WHOLE_ | 0.229 | 0.059 |
| Vascularity | EM_HARDEST_ | 0.321 | 0.007 |
| Pliability | EM_WHOLE_ | 0.590 | 0.000 |
| Pliability | EM_HARDEST_ | 0.589 | 0.000 |
| Thickness | EM_WHOLE_ | 0.279 | 0.020 |
| Thickness | EM_HARDEST_ | 0.366 | 0.002 |
| Pain | EM_WHOLE_ | 0.202 | 0.096 |
| Pain | EM_HARDEST_ | 0.296 | 0.014 |
| Pruritus | EM_WHOLE_ | 0.349 | 0.003 |
| Pruritus | EM_HARDEST_ | 0.460 | 0.000 |
