# Supplementary material for: Correlation between elastic modulus and clinical severity of pathological scars: a cross-sectional study
Source: Sci Rep. 2021 Dec 2;11:23324. doi: 10.1038/s41598-021-02730-0 (PMC8639709; doi:10.1038/s41598-021-02730-0)
Supplement: Supplementary file 2 — Supplementary Information 2. [file 41598_2021_2730_MOESM2_ESM.docx]

| Supplementary Table 2. Description of mVSS used in this study | |
| --- | --- |
| Scar assessment | |
| **Pigmentation(M):** | |
| 0 | Normal |
| 1 | Hypopigmented |
| 2 | Mixed |
| 3 | Hyperpigmented |
| **Pliability(p):** | |
| 0 | Normal |
| 1 | Supple - flexible with minimal resistance |
| 2 | Yielding - giving way to pressure |
| 3 | Firm - inflexible, not easily moved, resistant to manual pressure |
| 4 | Banding - rope- like issue that blanches with extension of the scar |
| 5 | Contracture - permanent shortening of scar, producing deformity or distortion |
| Height(H): | |
| 0 | Flat |
| 1 | <2mm |
| 2 | 2-5mm |
| 3 | >5mm |
| **Vascularity(V):** | |
| 0 | Normal |
| 1 | Pink |
| 2 | Red |
| 3 | Purple |
| **Pain:** | |
| 0 | Non |
| 1 | Occasional |
| 2 | Requiring medication |
| **Pruritus:** | |
| 0 | Non |
| 1 | Occasional |
| 2 | Requiring medication |
