# Supplementary material for: Correlation between elastic modulus and clinical severity of pathological scars: a cross-sectional study
Source: Sci Rep. 2021 Dec 2;11:23324. doi: 10.1038/s41598-021-02730-0 (PMC8639709; doi:10.1038/s41598-021-02730-0)
Supplement: Supplementary file 3 — Supplementary Information 3. [file 41598_2021_2730_MOESM3_ESM.docx]

Supplementary Table 3. Description of cdemographic and clinical variables

| **Variables** | **Method of assessment and grading** | **Ref.** |
| --- | --- | --- |
| Age on continuous scale | Assessed at dermatology clinic | in units of years |
| Duration on continuous scale | Time from the first discovery to the US examination | in units of years |
| Sex | Female  Male | female vs male |
| mVSS | Applied by two dermatologists with more than 5 years’ experience in evaluating scars, including pigmentation (0-3), vascularity (0-3), thickness (0-3), pain (0-2) and itchy (0-2) ^1^ | 0-18 |
| Scar length | Assessed by US examination | in units of mm |
| Scar width | Assessed by US examination | in units of mm |
| Scar thickness | Assessed by US examination | in units of mm |
| Supracutaneous height | Assessed by US examination | in units of mm |
| Intradermal height | Assessed by US examination | in units of mm |
| EMWHOLE | EM of the whole scar measured by US elastography in a longitudinal section | in units of kPa |
| EMHARDEST | EM of the hardest lesion in the scar measured by US elastography in a longitudinal section | in units of kPa |
| EMNORMAL | EM of the normal skin (1cm away from the scar) measured by US elastography in a longitudinal section | in units of kPa |
| Etiology | Assessed according to the clinical signs and symptoms Absence  Spontaneity  Inflammation  Operation  Chemical | spontaneity, inflammation, operation vs chemical |
| Treatment history | Assessed according to the clinical signs and symptoms Absence  Untreated  Triamcinolone  Laser  Operation  Mixed | untreated, hormone, laser, operation vs mixed |
| Scar location | Assessed according to the clinical signs and symptoms  Absence  Head and neck  Limbs  Chest and abdomen  Back | head and neck, limbs, chest and abdomen vs back |
| Echogenicity | Assessed by US examination according to the inner echo of the scar  Uniform echo  Mixed echo | uniform echo vs mixed echo |
| Boundary | Assessed based on the border between scar and the surrounding normal dermis at US examination  Clear  Unclear | clear vs unclear |
| Infiltration level | Assessed by US examination according to the skin layer of scar involvement  Dermis: only dermis infiltrated by the scar  Subcutaneous fat layer: subcutaneous fat layer and dermis infiltrated by the scar | dermis vs subcutaneous fat layer |
| Subclinical fistulous tracts | Assessed by US examination  Absent  Present | absent vs present |
| Calcifications | Assessed by US examination  Absent  Present | absent vs present |
| Blood flow type | Assessed based on the type of blood flow by US examination  Absent: no blood flow signal found in the scar  Vein: only vein found in the scar  Artery and vein: artery and vein found in the scar  Artery: only artery found in the scar | absent, vein, artery and vein vs artery |
| Blood flow distribution pattern | Assessed based on the location of vessel by US examination  Absent: no blood flow signal found in the scar  Central type: only blood flow signal found in the central area of the scar  Mixed type: blood flow signal found in the central and peripheral area of the scar  Peripheral type: only blood flow signal found in the peripheral area of the scar | absent, central type, mixed type vs peripheral type |
| Adler | According to Adler's method^2^, assessed by US examination.  0: no blood flow signal found in the scar  I: 1-2 punctate or rod like tumor vessels found in the scar  II: 3-4 punctate vessels or a long blood vessel penetrating into the lesion (its length could be close to or exceed the radius of the scar)  III: more than 5 punctate vessels or 2 longer vessels found in the scar | 0, I, II vs III |

1.Oliveira, G.V. *et al.* Objective assessment of burn scar vascularity, erythema, pliability, thickness, and planimetry. *Dermatologic surgery : official publication for American Society for Dermatologic Surgery [et al.]* **31**, 48-58 (2005).

2.Adler, D.D., Carson, P.L., Rubin, J.M. & Quinn-Reid, D. Doppler ultrasound color flow imaging in the study of breast cancer: preliminary findings. *Ultrasound in medicine & biology* **16**, 553-559 (1990).
